# Access to care for non-communicable diseases in Mosul, Iraq between 2014 and 2017: a rapid qualitative study

**DOI:** 10.1186/s13031-018-0183-8

**Published:** 2018-12-29

**Authors:** Louisa M. Baxter, Manal Shams Eldin, Ali Al Mohammed, Malika Saim, Francesco Checchi

**Affiliations:** 1 Faculty of Epidemiology and Population Health, London School of Hygiene and Tropical Medicine, London, UK; 2 Epicentre, 8 rue Saint-Sabin, Paris, France; 3 Médecins Sans Frontières Operational Centre Paris, Paris, France

**Keywords:** Non-communicable diseases, Hypertension, Diabetes, Access to healthcare, Conflict, War, Humanitarian, Iraq

## Abstract

During June 2014 to April 2017, the population of Mosul, Iraq lived in a state of increasing isolation from the rest of Iraq due to the city’s occupation by the Islamic State group. As part of a study to develop a generalisable method for estimating the excess burden of non-communicable diseases (NCDs) in conflict-affected settings, in April–May 2017 we conducted a brief qualitative study of self-reported care for NCDs among 15 adult patients who had fled Mosul and presented to Médecins Sans Frontières clinics in the Kurdistan region with hypertension and/or diabetes. Participants reported consistent barriers to NCD care during the so-called Islamic State period, including drug shortages, insecurity and inability to afford privately sold medication. Coping strategies included drug rationing. By 2016, all patients had completely or partially lost access to care. Though limited, this study suggests a profound effect of the conflict on NCD burden.

**Electronic supplementary material:**

The online version of this article (10.1186/s13031-018-0183-8) contains supplementary material, which is available to authorized users.

## Background

Between June 2014 and July 2017, Mosul, Iraq was completely or partly occupied by the so-called Islamic State (IS). From 17 October 2016, as Iraqi forces and allies gradually reconquered the city, Mosul’s population became increasingly exposed to fighting among the most destructive since 1945. Prior to the population of 1,137,000) from November 2016, as conditions deteriorated, about 800,000 people fled the city [1] out of a population of 1,137,000 [2].

Non-communicable diseases (NCDs) are increasingly recognised as drivers of excess morbidity and mortality in armed conflicts, particularly in the Middle East [3]. We report a rapid qualitative study of Mosul residents with NCDs, done to parameterise a mathematical model of NCD burden in crisis settings. We explored NCD care before and during IS occupation in 2014–2017.

## Methods

### Patients and data collection

We collected data between 30 April and 7 May 2017 within two camps (names omitted for security reasons) hosting displaced people from, respectively, Mosul East (largely reconquered by end January 2017) and Mosul West (more recent arrivals), where fighting persisted until July. Patients were seen by Médecins Sans Frontières outpatient clinics serving the camps. Given security constraints, heavy clinical workload and the need for rapid information for modelling, we asked a female and male medical doctor seeing patients of their same gender to sequentially recruit a fixed sample of 16 study participants, with a balanced gender ratio. Patients were eligible if aged ≥18y, diagnosed with hypertension, diabetes mellitus and/or chronic kidney disease before 2014, not presenting with signs, symptoms or a diagnosis of psychological trauma, and able in the clinician’s opinion to answer questions without experiencing distress.

Clinicians offered study participation after the consultation, obtained written informed consent and interviewed patients using a brief (10–20 min long) semi-structured questionnaire (Additional file 1). They asked first about patients themselves, then about others in Mosul with their medical condition, inquiring about year-on-year changes. Extensive notes were taken in Arabic during each interview and a bilingual staff member translated them into English.

### Analysis

A framework approach was used to summarise interviews into key themes and sub-themes, while considering evidence on events in Mosul from available public sources. Themes were then mapped onto O’Donnell’s (2007) four dimensions of access, namely availability, geographic accessibility, affordability and acceptability [4] (Additional file 1). Access to care has been conceptualized in various ways, with service utilisation or ‘realised access’ often used as a surrogate of ‘true access’ [5].

We used content analysis to identify the key themes. Firstly, we summarised interviews to theorise about the factors that impacted NCD care. This created a set of initial codes that represented descriptive characteristics of NCD care, e.g. rationing treatment. Secondly, we developed conceptual codes that represented potential causal factors that might impact care.

## Results and discussion

Fifteen patients were recruited (Table 1). The most common NCD was diabetes (12 patients); of three patients using insulin, one identified as having type 1 diabetes. Nine patients had hypertension and six patients had both NCDs. All patients had left Mosul between October 2016 and March 2017.

**Table 1:** Participant characteristics

Diagnosis	Male (*n* = 8)	Female (*n* = 7)	Total (*N* = 15)
Hypertension	3	6	9
Diabetes mellitus	7	5	12
Hypertension and diabetes mellitus	2	4	6

### Accessibility of services

**Fig. 1:**
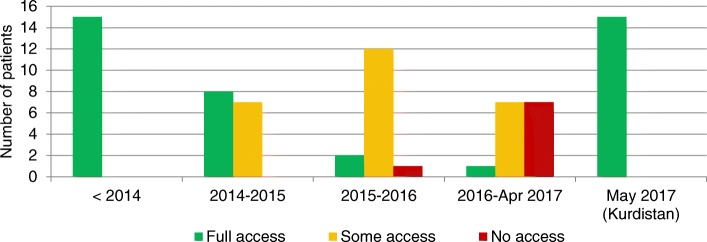
Number of patients (*N* = 15) reporting disrupted access to care, by period of time

Figure 1 shows self-reported access to NCD care (full access implies that the patient did not mention any disruption). Before 2014, all patients reported having had access to treatment and follow up. Seven reported receiving care from both the public (government hospitals, primary care clinics, pharmacies) and the private sector, six from the public sector only and three from private sources only.

All patients reported that their access to services had deteriorated during the occupation period, with many reporting no access whatsoever. The period 2016 to 2017 saw the worst access. By March 2017 only one patient (living on a farm outside Mosul) reported undisrupted care. All respondents commented that their care had now resumed at the MSF clinic.

The most frequently mentioned access barriers were cost (*n* = 15) and availability of drugs and clinical care (n = 15), followed by insecurity (*n* = 7) and transportation issues (*n* = 5). By early 2017, all patients were using the private sector instead of, or to supplement government services. The main reason given for this was limited drug availability in government facilities. One respondent commented that after 2014 “[the] only good access was at private hospitals or pharmacies because at government hospitals (there was) no treatment left”; another reported that the healthcare situation deteriorated as “the private pharmacies were slowly closing.” One patient noted a reason for reduced access was that hospitals were only seeing emergency cases. Security barriers mentioned included road closures, inability to pass through areas of the city controlled by the Islamic State, fear of attacks and roadside bombings.

Patients did not report suffering adverse events following treatment interruption, but one stated that her father had died due to complications of hypertension and ischaemic heart disease, while another reported a friend’s death from diabetes complications; respondents attributed both these deaths to problems with accessibility of healthcare.

### Availability of services

The commonest reported cause of NCD treatment interruption was inability to procure drugs. While drug availability diminished in both public and private sectors, the impact was greater in government clinics. Five patients rationed their drugs due to shortages and high cost, and two reported using only herbal medication for the last six months due to supply problems. Insulin availability appeared particularly affected in both public and private sectors. One patient reported that by 2015, government clinics were only prescribing sufficient insulin for ten days at a time; patients would then need to seek a repeat prescription. Four patients alluded to the absence of clinical staff in the public and private sector, and that this was worse in government facilities.

### Affordability of services

Before 2014, only one patient mentioned user fees in the public sector and noted that these were small and seemingly unrelated to diagnosis or services required. Most patients using the private sector did so primarily to buy medication: one patient stated that the drugs (anti-hypertensives and statins) required were not available in the public sector; however, it is unclear if for the others private care-seeking reflected drug unavailability or preference, e.g. for branded medicines. Two of the three patients using insulin said they purchased it from private sources.

After 2014, drug affordability diminished in both sectors and three patients mentioned that drugs and services that had been free prior to 2014 were now only available for a fee, one patient noting that government services were available “only if you had friends” working there. Four patients reported having to stop treatment due inability to purchase medications, and another sold his furniture, home and motorbikes to pay for care. Two patients explicitly mentioned differential access to care according to wealth; one noted that “everyone was facing the same issues for sure, those who had a house were better for sure”.

### Acceptability of services

One patient reported that managing her NCDs became less important to her as insecurity worsened: “There was war and shells and bombing so we didn’t even know what to do and never cared for our health, we just wanted to stay alive”. This may indicate that in some cases, even when services were available, patients were less likely to use them given overriding safety concerns. They may have adopted a health belief model that made chronic disease management less worthwhile and going without care acceptable given the larger existential threat. Only two patients discussed lifestyle modifications for their illness: both reported being unable to do this during the conflict. Five patients stated that stress made their illness difficult to control.

Most comments on clinical staff concerned non-availability rather than issues around relationships; however, one respondent did note that a reason she stopped attending the government clinic was increased staff rudeness and “aggressiveness”.

### Study limitations

A limitation of our study is the small sample size. Although in qualitative studies sample adequacy may be a more useful metric than sample size, further themes could have emerged in a more representative sample. Moreover, our non-stratified age sampling may affect generalisability. Although we found consistent themes across ages and genders, broader sampling would have been needed to fully illuminate barriers and facilitators for NCD care. We did not collect patients’ ages and as such responses may not represent the experience of older or younger NCD patients. Moreover, our sample does not provide a robust estimate of population coverage of NCD care.

A further limitation is that only patients able to access MSF clinics were recruited, There may be differences between these patients and those who do not access this care. Patients were recruited from only two sites displaced patients elsewhere may have had a different care experience. Generally, patients from Mosul West would have experienced a more prolonged and severe siege, with extensive damage to health services due to dense urban layout and a change in military tactics.

The use of physicians to both provide care for and interview patients may have affected how patients reported services, particularly their current care. Self-reporting itself may also introduce bias due to challenges with recall.

The study only collected data from patients with diabetes or hypertension; while this may have reflected convenience of recruitment, it is likely that these NCDs were the most prevalent among presenting patients. It seems likely that disruption of services and drug supply would have affected other NCDs in a similar way, though patients with other conditions, e.g. chronic obstructive pulmonary disease and chronic kidney disease may have had a higher risk of death if unable to access regular therapy such as dialysis or oxygen, and may therefore have been less likely to either escape Mosul alive, or present as outpatients.

## Conclusions

Whilst small, our study contributes to increasing documentation of the health consequences of occupation and war in Mosul and elsewhere in Iraq [6–10], and the effects on NCD care specifically [11–13].

Access to, availability, affordability and acceptability of healthcare services for NCDs in Mosul were adversely affected by conflict, siege and military occupation. While it is arduous to translate these findings into public health impact, we believe that, given the very high baseline burden of NCDs in Iraq, substantial excess morbidity and mortality among patients with NCDs may have occurred in Mosul.

Future studies in this area could address limitations outlined above**,** for example by exploring experiences of patients across different age groups and other NCDs, e.g. how young children and younger adults with haemoglobin disorders (e.g. thalassemia, highly prevalent in the Middle East) may have been affected. A further area of research is the NCD care experience of people with mental health problems. The adoption of alternative methods of disease management (e.g. lifestyle changes, herbal therapies, traditional remedies) should also be better documented in conflict settings. Given the progression of NCDs in the absence of treatment, exploring access to tertiary level care such as dialysis, chemotherapy, radiotherapy and palliative care would offer important insights.

It is vital for humanitarian actors to understand the impact of conflict on patients with NCDs in order to draft policy design appropriate interventions. Better integration of NCD care into routine public health programs, including the availability of NCD medications and services as part of standard emergency health bundles, modified guidelines and standards appropriate to disease management in conflict afflicted settings, and health information systems that support chronic disease data collection and monitoring at the patient level, could all contribute to improve NCD outcomes. Humanitarian actors should also consider the socio-economic barriers to NCD care arising from protracted conflict periods, and address these through social protection measures.

## Additional file

Additional file 1: Interview questions and identified barriers to accessing healthcare. (DOCX 23 kb)
