# Supplementary material for: Access to care for non-communicable diseases in Mosul, Iraq between 2014 and 2017: a rapid qualitative study
Source: Confl Health. 2018 Dec 29;12:48. doi: 10.1186/s13031-018-0183-8 (PMC6311030; doi:10.1186/s13031-018-0183-8)
Supplement: Supplementary file 1 — Interview questions and identified barriers to accessing healthcare. (DOCX 23 kb) [file 13031_2018_183_MOESM1_ESM.docx]

**Additional file 1**

**Table S1. Interview questions**

| **Interview question** | **Specific probes** |
| --- | --- |
| 1. Please think back to your time living in Mosul, before and after what happened in June 2014. Can you talk about your experience living in the city as a patient with a (hypertension, diabetes etc.) diagnosis? |  |
| 1. To what extent were you able to access treatment for your condition before June 2014? |  |
| 1. What about since June 2014? To what extent were you able to access treatment after that time? Can you talk about what made it easy and what made it hard to do so? | - How did the situation evolve for you? - How was the availability of treatment in 2014? - How did the situation evolve for you? - How was the availability of treatment in 2015, if you were still living in the city? - How did the situation evolve for you? - How was the availability of treatment in 2016, if you were still living in the city? - What about this year? |
| 1. What do you think about other people’s experiences? Thinking generally about people in Mosul, how do you think other people with a similar medical problem to yours managed? | - How did the situation change over time, since 2014? - How did you or other people obtain medicine for your condition after June 2014? |
| 1. Is there anything else that you would like to say about this topic or of your experience? |  |

**Table S2. Overview of identified access barriers to health care**

| **Dimension** | **Supply side** | **Demand side** |
| --- | --- | --- |
| **Accessibility**: | Service location  Non-existent services | Indirect costs to household (transport)  Means of transport available |
| **Availability:** | Unqualified staff, absent staff, staff motivation  Opening hours, waiting time  Drugs and other consumables  Non-integration of health services, Lack of opportunity (exclusion from services)  Late or no referral | Information on health care service/providers’ education |
| **Affordability** | Costs and prices of services, including Informal payments  Private-public dual practices | Household resources and willingness to pay  Opportunity costs  Cash flow within society |
| **Acceptability** | Complexity of payment system, Inability of patients to pay  Staff interpersonal relations including trust | Household expectations  Low self-esteem and assertiveness- health literacy  Community and cultural preferences  Stigma  Health literacy and illness and health beliefs  Knowledge of health care  Concepts of agency, rights and capacity to choose to seek health care  Knowledge about health care options, participation and involvement of the patient in treatment decision-making |

Adapted from Peters et al. [1], Ensor and Cooper [2], Levesque et al. [3] and O’Donnell [4].

**References**

1. Peters DH, Garg A, Bloom G, Walker DG, Brieger WR, Rahman MH: **Poverty and access to health care in developing countries**. *Ann N Y Acad Sci* 2008, **1136**:161-171.

2. Ensor T, Cooper S: **Overcoming barriers to health service access: influencing the demand side**. *Health Policy Plan* 2004, **19**(2):69-79.

3. Levesque JF, Harris MF, Russell G: **Patient-centred access to health care: conceptualising access at the interface of health systems and populations**. *Int J Equity Health* 2013, **12**:18.

4. O'Donnell O: **Access to health care in developing countries: breaking down demand side barriers**. *Cad Saude Publica* 2007, **23**(12):2820-2834.
